# A Pencil-Drawn Electronic Tongue for Environmental Applications

**DOI:** 10.3390/s21134471

**Published:** 2021-06-29

**Authors:** Dmitry Kirsanov, Subhankar Mukherjee, Souvik Pal, Koustuv Ghosh, Nabarun Bhattacharyya, Rajib Bandyopadhyay, Martin Jendrlin, Aleksandar Radu, Vladimir Zholobenko, Monireh Dehabadi, Andrey Legin

**Affiliations:** 1 Institute of Chemistry, Mendeleev Center, St. Petersburg State University, Universitetskaya nab. 7/9, St Petersburg 199034, Russia; dehabadi16752@gmail.com (M.D.); a.legin@spbu.ru (A.L.); 2 Laboratory of Artificial Sensory Systems, ITMO University, Kronversky pr. 49, St Petersburg 197101, Russia; 3 Agri and Environmental Electronics (AEE) Group, Centre for Development of Advanced Computing (C-DAC), Sector—V, Salt Lake, Kolkata 700091, India; subhankar.mukherjee@cdac.in (S.M.); souvipal@gmail.com (S.P.); koustuv02@gmail.com (K.G.); nabarun.bhattacharya@cdac.in (N.B.); 4 Department of Instrumentation & Electronics Engg, Jadavpur University, Salt Lake Campus, Block LB, Sector III, Kolkata 700098, India; rajib.bandyopadhyay@jadavpuruniversity.in; 5 Lennard-Jones Laboratories, Birchall Centre, Keele University, Keele, Staffordshire ST5 5BG, UK; m.jendrlin@keele.ac.uk (M.J.); a.radu@keele.ac.uk (A.R.); v.l.zholobenko@keele.ac.uk (V.Z.)

**Keywords:** electronic tongue, multisensor system, potentiometric sensors, zeolite, environmental monitoring

## Abstract

We report on the development of a simple and cost-effective potentiometric sensor array that is based on manual “drawing” on the polymeric support with the pencils composed of graphite and different types of zeolites. The sensor array demonstrates distinct sensitivity towards a variety of inorganic ions in aqueous media. This multisensor system has been successfully applied to quantitative analysis of 100 real-life surface waters sampled in Mahananda and Hooghly rivers in the West Bengal state (India). Partial least squares regression has been utilized to relate responses of the sensors to the values of different water quality parameters. It has been found that the developed sensor array, or electronic tongue, is capable of quantifying total hardness, total alkalinity, and calcium content in the samples, with the mean relative errors below 18%.

## 1. Introduction

The fast global development of urban, industrial, agricultural, and transport technologies has posed a significant threat to the environment. These sectors discharge huge quantities of gaseous, liquid, and solid waste every day. The waste may be discharged directly to the environment, such as dumping grounds, open air, lakes, rivers, and, ultimately, oceans, without proper treatment and decontamination. Of course, the rules of waste disposal are often rather strict, and most enterprises follow them. However, an adequate chemical analysis of the environmental samples is a bottleneck in the management chain. The variability and complexity of contaminants, as well as possible unpredictable interactions and concerted influence of these components on the environment and living beings, make it imperative to develop effective chemical sensors for real-life environmental analyses. 

Effective monitoring systems of water pollution should exhibit characteristics as reasonable accuracy, high stability and reproducibility, simplicity of analysis, cost-effectiveness, and ease of fabrication. Such monitoring systems must be fast enough to enable speedy detection of contaminants, providing the basis for effective actions. Many traditional laboratory methods, such as inductively coupled plasma optical emission spectroscopy (ICP-OES), and gas and liquid chromatography (GC and LC), especially coupled with mass-spectrometric detection, can be very accurate and sensitive but require long and tedious calibration. These, and many other methods, assume exhaustive sample preparation sequences and complex analytical procedures. In addition, these methods require expensive instrumentation, costly reagents, and expert operators to conduct the analysis. These drawbacks make many conventional detection methods hardly useful for online and onsite environmental monitoring.

Chemical sensors seem to be an obvious alternative to highly sophisticated laboratory-based instruments. Such sensors are numerous and well developed, but their application in a multicomponent environment can easily appear quite cumbersome due to the complexity and uncertainty of the analytical signal they produce. Nevertheless, relatively simple chemical sensors are probably the most prospective and realistic way to achieve a wide-scale environmental analysis through distributed sensor networks. In this respect, the development of simple and inexpensive devices capable of chemical analysis is a very timely problem. The development of paper-based analytical tools is one of the perspective directions in such developments. Curiously, paper-based chemical sensors are probably the oldest known sensing devices for aqueous media analysis. The so-called litmus paper, widely used for semi-quantitative pH determination, is well known for over two hundred years, while the chemistry behind it is even much older. 

In the “modern history”, it is usually stated that the first paper-based sensors were developed in 1956, to monitor the glucose level in urine [1]. However, the first documented evidence of the paper-based technology dates back to 1902, to a patent, filed by Dieterich (US691249A). The later developments of paper-based sensors were by and large concerned with different biosensors, especially for simple point-of-care applications. The most common paper-based sensors are pregnancy test kits, e.g., References [2,3]. Such detection kits are based on the immunoassay method comprising a sample pad, a reagent pad, and a test line. The sample pad receives the tested sample, the reagent pad contains antibodies conjugated to target antigen-specific signal indicator, and the test line captures the antibodies immobilized on the surface. The signal produced in such devices is generally a color standing for the qualitative analysis (Yes-No type) of a sample.

A range of biomedical applications of paper-based devices were enabled by their portability, low-cost, low-volume, disposable design and simplicity of the analytical procedures. One can mention, e.g., diagnostic of liver function via analysis of serum transaminase enzymes [4], blood type by detecting red blood cell antigens [5], a wide range of biomedical analytes, such as cancer and tumor markers, mycobacterium tuberculosis, HIV antibodies [6], infection decease markers [7], and many others. Enzyme-based biosensors utilizing electrochemical and optical transduction [8,9] are widespread due to the simplicity of operation, compactness, and high selectivity. 

It must be mentioned that many enzyme-based biosensors suffer from certain inherent disadvantages, such as long and complicated production processes from limited natural sources [10,11], limited shelf life, and specific storage requirements [12]. This leads to a price increase of such devices, diminishing the advantages of the paper as a substrate.

Non-enzymatic paper-based sensors have recently been explored and developed, to replace enzymes with more stable, reproducible, and simple materials [13]. Non-enzymatic sensors commonly employ nanostructured materials promoting catalytic reactions or using other transduction mechanisms, such as changes in optical properties [14]. Noble metals (Au, Ag, Pt) [11,15], carbon-based materials (CNTs, quantum dots) [16], and metal oxides (ZnO, NiO, MnO_2_) [17] were suggested for such sensors, mostly due to their remarkable catalytic activity. The obvious need for simple and reproducible non-enzymatic reactions to be employed, however, limits the variety of such sensors. 

It must be pointed out that the substrate is of utmost importance for paper-based sensors. Besides the selection of the most suitable paper material, further modification or patterning of the paper substrates must be carried out in most cases. There is a requirement for the fabrication of microchannels, electrodes, specific layers, or structures on paper materials. Several chemical or physical techniques are available for such modifications. Such methods as plasma treatment [18], photolithography [19], inkjet printing [20], screen printing [21], wax treatment [22], and embossing [23] were suggested for the paper sensors. The idea behind all these methods is to develop a fabrication protocol for a microfluidic structure on paper, enabling various methods for sample preparation, pre-treatment, targeted delivery and multi-step detection. Microfluidic methodologies are making the resulting paper-based sensors more diverse but more complicated, as well. 

Ink-jet printing can be considered as an outstanding methodology, which is widely applied to the fabrication of paper-based sensors [24]. However, most ink compositions used for such sensors are either aqueous or are based on polar organic solvents, ultimately producing water-soluble layers not useful for liquid media analysis. Not surprisingly, ink-jet materials have been mainly suggested for a variety of physical sensors [25]. Although ink-jet chemical sensors were proposed a long time ago [26], they are still mostly applied to gas analysis [24,27] (and references therein).

Applications of paper-based sensors for environmental analysis of aquatic samples are rather scarce. While the idea of paper-based sensor analysis of real-life aqueous samples was put forward over ten years ago [28,29], the publications in this domain are still infrequent. Notable paper-based sensors have been proposed for the detection of few selected pollutants in real environmental waters, such as organophosphorus pesticides [22], heavy metals [29,30,31], or phenolic compounds [32]. However, the suggested sensors hardly displayed sufficient sensitivity or LOD for effective environmental application. Furthermore, they are based on bioreactions and microfluidic structures that are making their fabrication a precise high-tech process.

Recently, a new approach towards the development of very simple and cost-effective sensors has been suggested [33]. It relies on the employment of zeolite-based “pencils” that can “draw” sensor lines on polymeric support, thus yielding simple potentiometric ion-sensitive devices (Ion Sensitive Pencils (ISPs)) capable of quantitative analysis of aqueous samples. The authors argue that compression of different zeolites with graphite result in a tool (pencil) that can be used to produce electrodes that do not require pre- and post-measurement handling. ISPs can be stored in a dry place and electrodes can be produced based on the demand. The extreme simplicity of ISPs and produced sensors and sensor handling allow recruitment of non-trained personnel for analysis, thus further reducing cost of analysis. Zeolites were suggested for sensor applications over two decades ago, e.g., References [34,35]. Since then, zeolites have been largely applied in gas sensors [36] (and references therein), whereas their applications for aqueous media are scarce. Their ion exchange property and the ability to withstand pressures needed to produce ISPs (4+ tonnes as shown in the original publication [33]) were the key driver of their application in ISPs. However, their selectivity is inferior to classical ionophores so the authors have suggested that the power of ISPs may lay in their application in multisensor arrays. 

In this study, we aim to develop this sensing platform further by combining it with the “electronic tongue” multisensor concept. “Electronic tongues” are arrays of cross-sensitive chemical sensors assisted with chemometrics tools for multivariate data processing [37]. These devices have been validated as efficient analytical instruments in numerous applications [38,39]. They are also actively studied as convenient tools for environmental applications [40,41]. In order to assess the applicability of “pencil-drawn” sensor arrays for real-life analytical analyses, we have constructed a zeolite-based multisensor system and employed it for the potentiometric measurements in the analysis of real surface waters sampled from two Indian rivers in West Bengal. The same samples were also characterized by conventional analytical tools with respect to the general water quality parameters, and the correlations between the sensor response and conventional methods have been examined.

## 2. Materials and Methods

### 2.1. River Water Samples 

The samples for analysis (100 in total) were taken from two rivers in West Bengal, India. The Mahananda River was sampled in 14 different locations with geographical coordinates ranging in 25.1–26.8° N and 87.8–88.4° E. Each point was sampled twice—in the morning (6:00–9:30 local time) and the afternoon (13:00–15:00). Thus, the total number of samples from the Mahananda was 28. Several generalized water quality parameters were measured immediately after sampling on site. TDS (total dissolved solids) varied from 18 to 61 ppm (average 41), pH 6.63–8.37 (average 7.18), and electrical conductivity was 36–122 μS/cm (average 82).

The Hooghly River was sampled in 24 locations with the coordinates in the range 22.1–24.8° N and 87.9–88.5° E. In this case, the samples were taken three times a day—in the morning (6:00–9:30), in the afternoon (13:00–14:30), and the evening (16:50–18:00). The total number of samples from Hooghly was 72. TDS was changing between 124 and 2533 ppm (mean 286), pH 6.98–8.11 (mean 7.79), and electrical conductivity varied between 248–5066 μS/cm (mean 572). It can be seen that, according to these general parameters, the Hooghly River appears to be more contaminated.

### 2.2. Sensor Preparation

Sensors were prepared and thoroughly characterized according to a procedure reported in our previous publication [33]. Briefly, synthetic graphite powder (<20 μm, Sigma-Aldrich) and a zeolite (Table 1) were mixed in 60:40 weight ratio in a ball mill. A uniform mixture was pressed into a pellet using a 13 mm pellet die (Specac) by a hydraulic bench press (KENNEDY Hbp010). A series of experiments measuring response slopes of sensors utilizing different graphite:zeolite ratios and the press loads enabled us to identify composition of 60:40 wt.% and the load of 4 tonnes as optimal, since such sensors exhibited near-Nernstian slopes [33]. 

**Figure 1:**
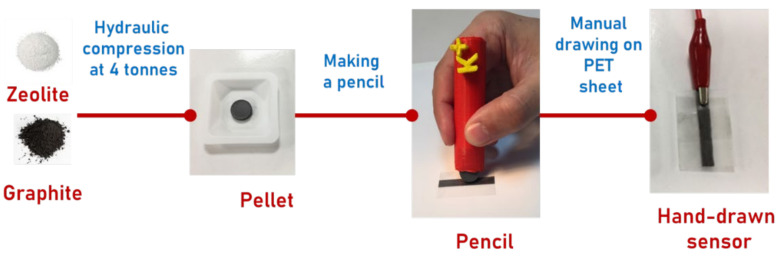
Sensor preparation scheme.

The prepared pellet (a.k.a. ISP lead) was inserted into a pencil clutch prepared using a 3D printer (PLA filament, Wanhao Duplicatior 4S) to obtain Ion-Sensing Pencil (ISP). Obtained ISP was used to hand-draw a line onto the PET sheet etched with the aluminium oxide (grit 240) for 30 s to provide a rough surface with enhanced porosity and improved adhesion of ISP lead. The sensor was obtained by abrasion until the resistance of the drawn line was lower than 3.0 kΩ, as suggested previously [44]. The overall scheme of the sensor preparation is given in Figure 1. In total, twelve zeolites were used (Table 1), and each sensor contained only one type of zeolite. Further on, these sensors are encoded as s1–s12, correspondingly.

### 2.3. Potentiometric Measurements

Potentiometric measurements with “pencil drawn” sensor array were performed in the following galvanic cell:

Cu|Ag|AgCl, KClsat|sample solution|zeolite layer|Cu.

**Figure 2:**
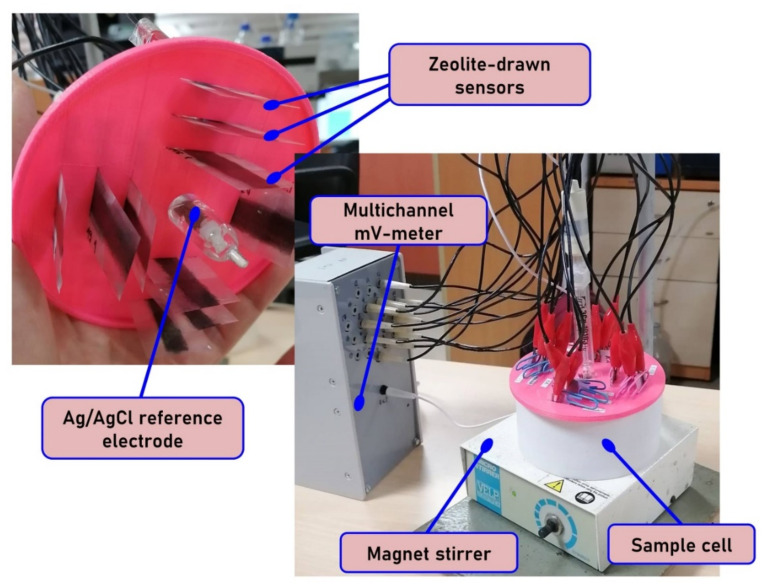
Experimental set-up with pencil-drawn sensors.

The visual appearance of the measuring set-up is given in Figure 2. The measurements were carried out using multichannel mV-meter KHAN-10 (Sensor Systems LLC, St. Petersburg, Russia) connected to a PC for data acquisition and processing. The electromotive force (EMF) was measured with 0.1 mV precision against the standard reference silver/silver chloride electrode (Izmeritelnaya Tekhnika, Moscow, Russia). 

The measurements in the river waters were performed using ~70 mL of sample poured into a Teflon measuring cell. The duration of the measurement for each sample was fixed at 3 min. Sensor potentials were registered every 12 s within this period. The recorded EMF values were collected into a data file for further processing. The last three readings in 3 min measurement were averaged for multivariate modeling. After each sample, the sensors were washed with 3 portions of freshly distilled water, for 2 min each. 

### 2.4. Reference Analysis

River water samples were characterized in a certified laboratory with respect to the total hardness, total alkalinity, and the content of sodium, potassium, magnesium, and calcium, using standard analytical tools.

*Total hardness*. The presence of bicarbonate, chlorides, and sulfate salts of calcium and magnesium ions in water is measured in terms of the water hardness. A higher hardness of water is unhealthy for consumption for drinking, bathing, and washing and forms scales in boilers. The estimation is based on complexometric titration and done by the standard ethylene di-amine tetra-acetic acid (EDTA) method.

The total hardness of water was measured using Eriochrome Black-T (EBT) indicator. Upon addition, the EBT forms a wine-red colored complex in the presence of these cations. On addition of EDTA, EBT forms a steel-blue complex in the presence of an ammonia buffer [45]. 

*Total Alkalinity.* This is the capacity of a solution to neutralize the acids present. Bicarbonates, carbonates, and hydroxides remove the hydrogen ions and lower the acidity of solution. It is conventionally measured by a double endpoint titration method using a burette or a digital titrator according to the EPA standard protocol. The reagent, sulfuric acid, was added stepwise to determine the endpoint of titration until pH reached 4.2 (the amount of acid used is equivalent to the alkalinity of the solution). The collection of the samples using a burette was carried out according to the standard APHA, 1992 method [46,47]. 

*Determination of specific ions in the water samples.* The recommended tests have been performed at National Accreditation Board for Testing and Calibration Laboratories (NABL) accredited laboratory. The conventional standard method for the detection of ionic species in solution is done by Ion Chromatography (IC). Highly pressurized columns for separating different ions were employed. The instrument used for analysis was Dionex™—ICS-6000 HPIC™ (ThermoFisher Scientific, Waltham, MA, USA). All the details on instrumental protocol are reported in Reference [48].

### 2.5. Data Processing Methods

Principal component analysis (PCA) was employed to obtain the maps of sample compositions and to assess the suitability of the multisensor system for discriminating the river waters with different level of contamination. PCA is an unsupervised dimensionality reduction method aiming to represent the samples characterized by multiple variables in a series of 2D plots (scores and loadings plots). These plots can be used to judge the similarity or dissimilarity of samples under study and the impact of particular variables on these differences. A clear tutorial on PCA can be found in Reference [49]. 

In order to assess the similarity in the variance structure of two matrices, we employed Tucker’s congruency coefficient, which is a standardized measure of proportionality of the elements in two matrices [50]. It was calculated as follows:

*ϕ* = *tr*(*T*_1_*T*_2_^*T*^)/*sqrt* (*tr*(*T*_1_*T*_1_^*T*^) *tr*(*T*_2_*T*_2_^*T*^),

where *T*_1_ and *T*_2_ are the score matrices obtained by PCA for two datasets (multisensor data and reference data) with the same number of rows. Tucker’s coefficient varies from −1 to +1. The number of columns (the number of PCs taken into account by corresponding PCA models) can be different, and it is recommended to attain the same level of the explained variance in two models; in our study, this value was set to 95%.

In order to relate the response of the sensor array with the concentrations of target analytes in the river water samples, we constructed a multivariate regression model using partial least squares (PLS) algorithm [51]. This tool aims to find the variance in the matrix of predictors (sensor responses) that would be correlated to the variance in the concentration vector. PLS is based on decomposition of ***X*** matrix (sensor responses) in the way similar to that of PCA, with the difference that the LVs (latent variables, analogues of PCs in PCA) are calculated according to the variance in ***X*** which is correlated to the variance in ***Y*** (concentrations of target analytes). These LVs are further employed to calculate regression coefficients ***B***—their values will depend on the number of LVs involved in the decomposition. The number of LVs is the main parameter in PLS, and it is important to choose it carefully as it will affect the precision of the regression modeling. If the number of LVs is too small, then the model does not describe the data completely, and it is called underfitted, the values of ***B*** will take into account not enough of the relevant variance in X, and the model precision will be insufficient. If the number of LVs is too large, then the regression coefficient will take into account the variance which is not related to the modeled property (the concentration of target analyte), but with some other source, like, e.g., instrumental noise. The resulted regression coefficients of the model would highlight the most important variables associated with the target parameter under study. 

The number of samples in the study was sufficient to implement test set validation; however, in order to avoid an uncertainty associated with the random selection of a single test set, a more rigorous validation procedure was employed. It was based on 10-fold segmented cross-validation with 10 different samples randomly chosen to serve as a test set 10 times, and the resulting figures of merit (squared correlation coefficient for “measured vs. predicted” plot and root-mean-squared error of prediction) were averaged over 10 runs for each of the modeled parameters. This type of validation is also referred to as Monte-Carlo cross-validation, and it provides for a thorough assessment of the model validity.

The quality of the constructed PLS models was evaluated using traditional prediction performance metrics, such as root mean-square error of cross-validation (RMSECV) (Equation (1)) and the respective coefficients of determination (R^2^).

(1) $$RMSECV=\sqrt{\frac{\sum_{i=1}^{k}{\left({\hat{y}}_{i}-y_{i}^{}\right)}^{2}}{k}},$$

where *y_i_* and ${\hat{y}}_{i}$ are known and predicted values of the target calibration parameter, and *k* is the number of samples in the validation set. 

Multivariate data processing was performed in R [52] using the *mdatools* package [53].

## 3. Results

### 3.1. Characterization

**Figure 3:**
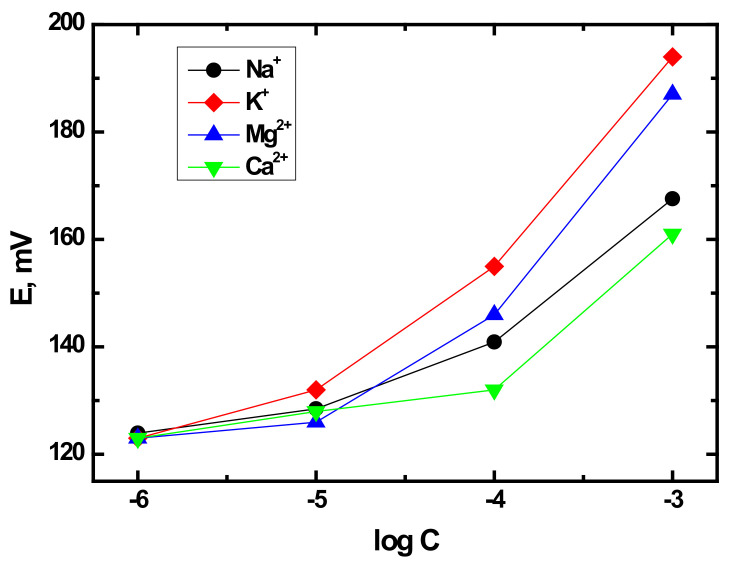
Potentiometric response curves for NaX zeolite sensor.

Initial characterization of ISPs revealed that their response behavior is analogous to classical ion selective electrodes (ISEs) [33]. This is not surprising given that ion exchange is the key response mechanism. Therefore, near-Nernstian responses of individual sensors is expected while using potentiometry as the analytical technique. Herein, initial assessment of the zeolite-based sensors was performed in the individual aqueous solutions of alkali and alkali-earth metals. As a typical example of the potentiometric response, Figure 3 shows the calibration curves obtained for NaX zeolite sensor in Na^+^, K^+^, Mg^2+^, and Ca^2+^ solutions, demonstrating that the sensor response varies between different cations. The values of sensor sensitivity calculated between 10^−5^ and 10^−3^ M for all studied sensors are reported in Table 2. Specific details of the experimental protocols and discussion of these results are available in Reference [33]. Briefly, sensors exhibit various degrees of agreement with near-Nernstian slopes, which is not surprising given that selectivity of zeolites is inferior to classical ISEs that rely on strong covalent interaction of the target analyte to a very selective ionophore. The origin of zeolites’ selectivity is more complex and relies on factors, such as zeolite channel structure, pore size, and Si/Al ratio in the zeolite structure [42]. However, most studies have been typically focused on the application of zeolites in catalysis. In fact, to the best of our knowledge, there is no available study of the influence of these factors on sensing purposes. This is a topic of research currently performed in our group to be reported in a forthcoming publication. 

### 3.2. Application

**Figure 4:**
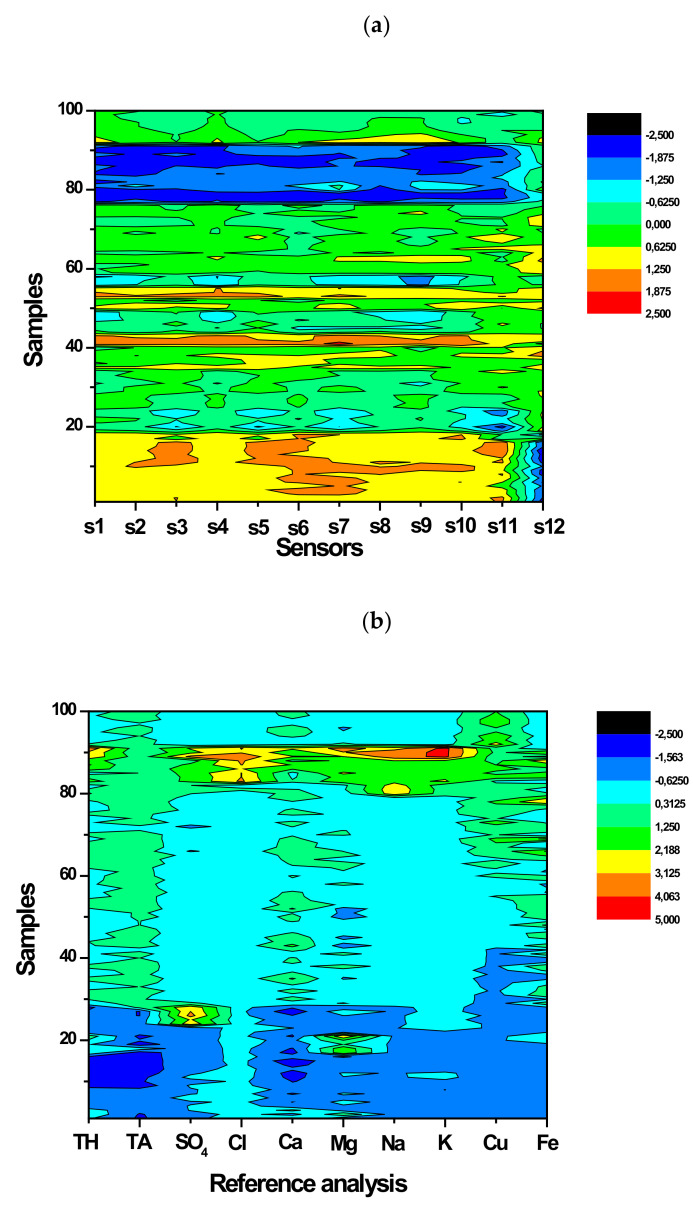
The heatmaps of the autoscaled data from the multisensor array (**a**) and the reference analytical methods (**b**). Samples 1–28 are from Mahananda River, and samples 29–100 are from Hooghly River.

Twelve “pencil-drawn” sensors were organized into a sensor array and the measurements were performed in real surface waters sampled from the rivers. The same samples were characterized using standard analytical techniques to assess chemical parameters of the water quality. The purpose was to investigate if it is possible to relate the response of such sensor array with certain water quality parameters. Figure 4 shows the heatmaps of the autoscaled potentiometric sensor responses (Figure 4a) and the autoscaled analyte concentration data from the reference methods (Figure 4b). Sensor encoding (s1–s12) corresponds to Table 2. Examples of potentiometric response and reference data are provided in the Supplemental Info (Tables S1 and S2). As autoscaling returns dimensionless units, the color map has the same dimensionless nature, and the absolute values of the resulted sensor response and reference data are encoded with color.

Inspection of the heatmaps reveals that the two datasets highlight similar differences in the chemical composition of river water samples. In this way, both the reference chemical analysis and multisensor analysis show that the group of samples from 1 to 20 is different from the rest (yellow color for sensor responses and blue for reference analysis), the same holds for the samples around 80 to 90 (compare the colors in the heatmaps; they differs from the rest in both maps — blue for the sensors and yellow/green for reference parameters). The reference methods heatmap also indicates that the Mahananda River has all chemical parameters lower than those for Hooghly (bottom part of the map). The data on average values of water quality parameters in two sampled rivers are provided in Section 2.1 and the difference between the rivers is in good agreement with the observed color patterns in Figure 4a. Taking into account that the samples were measured in a random order, this demonstrates the potential of the “pencil-drawn” multisensor array for capturing the patterns of general water quality utilizing a simple and cost-effective measuring procedure. 

**Figure 5:**
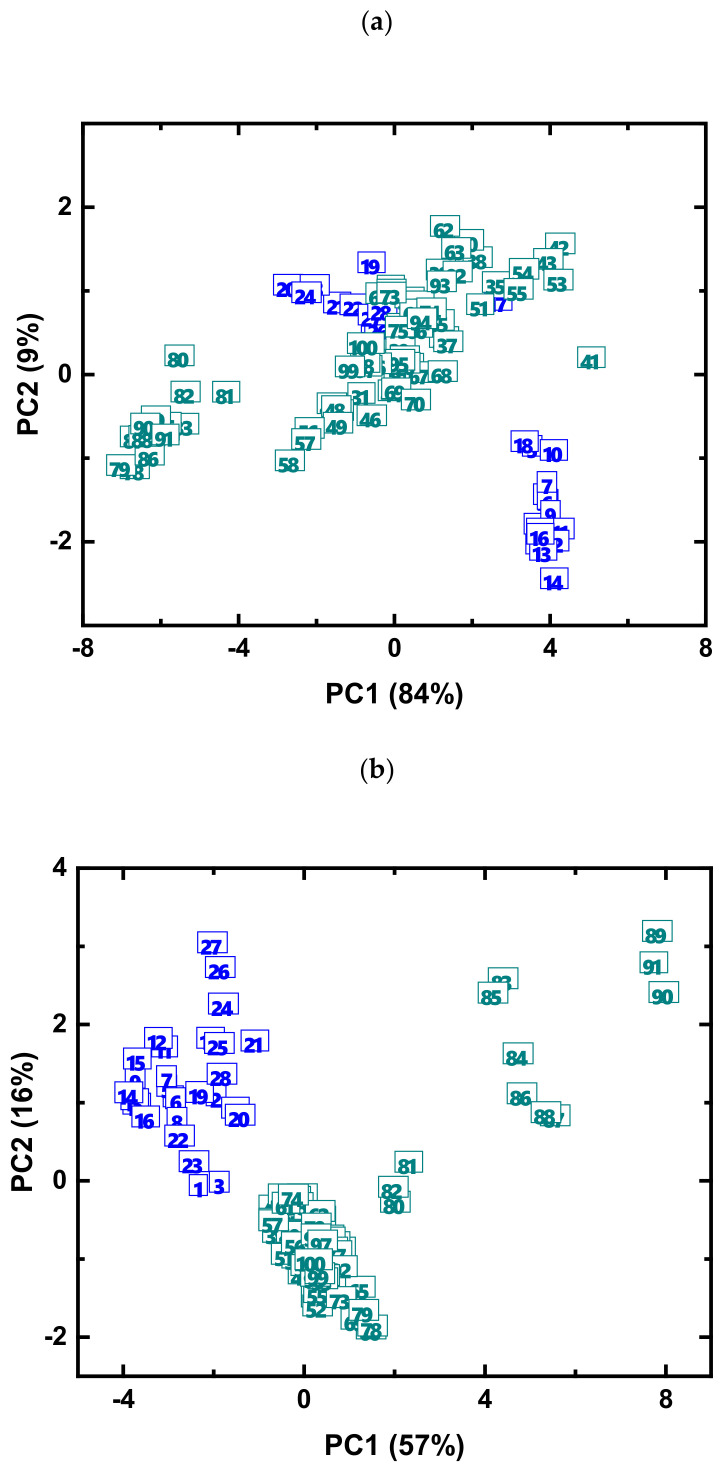
PC1-PC2 score plots for the multisensor array data (**a**) and the reference analytical methods (**b**). Samples 1–28 are from Mahananda River (blue numbers), and samples 29–100 are from Hooghly River (dark cyan numbers).

At the next step, the PCA score plots were calculated for both datasets (Figure 5). The objective of this PCA analysis was to confirm that the results of conventional analytical methods and the results from sensor array are comparable, and both tools allow capturing the same difference in the chemical composition of the samples.

Although capturing the different amount of the explained variance (93% for the multisensor arrays and 73% for the standard analytical techniques) and having a different structure in sample distribution, these PCA score plots reveal similar clustering patterns of the samples. The blue group of samples (from Mahananda river) forms two separate clusters in sensors score plot (Figure 5a) and a single cluster in the reference analysis score plot (Figure 5b). The overlapping of the cluster of samples from Mahananda river with the large cluster of samples from Hooghly river can be explained by the fact that sensor array conveys a somewhat different chemical information about the samples compared to reference analysis. At the same time the group of samples around 80–90 from Hooghly river is clearly separated in both PCA score plots. The two score matrices were employed to calculate the Tucker congruency (a measure of common variance in two matrices) coefficient between these matrices, and it was found to be −0.669, thus indicating a large amount of shared information between the two datasets.

**Figure 6:**
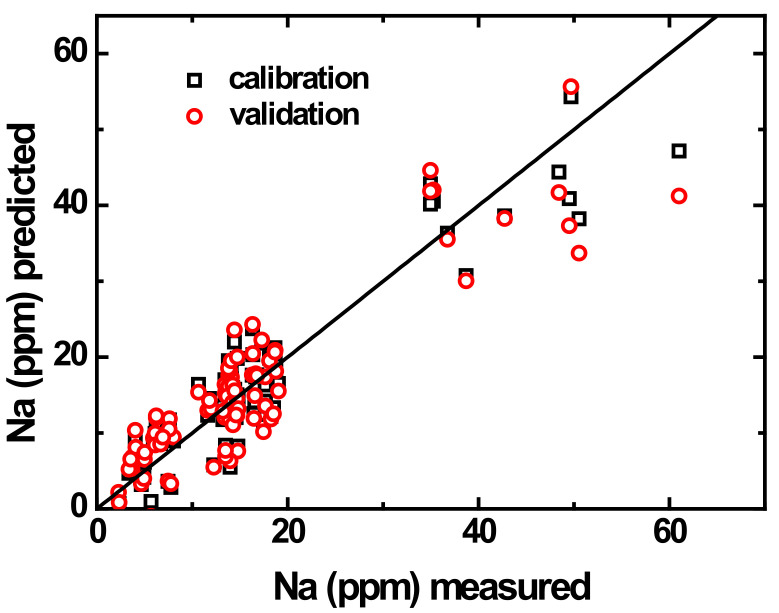
Measured versus predicted plot for 10-fold segmented cross-validation of the PLS model for prediction of sodium content.

The data evaluation step yielded promising results in assessment of pencil-drawn multisensor system potential for water analysis, and it was followed by the regression modeling using the PLS technique in order to explore the possibility of the numerical prediction of certain chemical parameters in river waters based on the response of the zeolite sensor array. Figure 6 shows a typical “measured vs. predicted” plot obtained in 10-fold segmented cross-validation of the PLS model intended for numerical prediction of sodium content. A reasonably high correlation can be observed in the data points. Table 3 shows the figures of merit of multivariate regression models obtained in the prediction of several water quality parameters. The range of the variable change is different for different parameters; thus, the inspection of the RMSECV values should be done with accounting for these differences. As a more convenient measure of model performance, we have also calculated mean relative errors (MRE) that can be considered directly, without the range.

There were no reasonably good models constructed for the rest of the reference values, such as sulfate, chloride, copper and iron content. The lack of correlation with anion content is expected due to the cationic sensitivity type of zeolite sensors. The quantification of heavy metals in river waters is not feasible with the designed system due to their comparatively low content, especially as free ions, in the natural water and complex river water matrix.

To summarize, the ability of the pencil-drawn multisensor system to differentiate the groups of samples based on their composition was clearly demonstrated by the following:

(1) the heatmaps for raw sensor responses and reference analysis demonstrate similar differences in color distribution for the samples from Mahananda and Hooghly rivers — this means that both approaches capture the same chemical information;
(2) PCA score plots for sensor responses and reference analyses are showing similar sample grouping — this means that both methods capture similar variance in the data;
(3) Tucker congruency coefficient between the matrices of sensor responses and reference analysis is found to be −0.669, thus indicating a large amount of shared information between the two datasets;
(4) the values of MRE for prediction of total hardness, total alkalinity, and calcium content are below 20%, which is a clear indication of analytical power of this extremely simple sensor array.

## 4. Conclusions

Based on the presented results, it can be concluded that a pencil-drawn sensor array is capable of a quantitative assessment of chemical composition in real river water samples with acceptable precision. The worst mean relative errors have been attained for sodium and magnesium, while the best precision has been observed for the integral quality parameters, such as total hardness and total alkalinity. Taking into account the ultimate simplicity of the proposed sensor array construction and very cost-effective preparation protocols, we believe this approach can be further elaborated towards other real-life applications in analysis of aqueous media. The authors are not aware of any other analytical instrument that would compete with the proposed system in terms of cost-efficiency but yet has been capable of simultaneous quantification of several water quality parameters in real surface waters (not an easy task even for conventional analytical tools). The achieved precision appears to be sufficient for alarm situation monitoring. 

## Figures and Tables

**Table 1:** Selected features of zeolites used in the multisensor array *.

Zeolite	Extraframework Ion	Structure Type	Si/Al Ratio	Origin (Supplier)
Na-X (Linde Type X)	Na^+^	FAU (Faujasite)	1.3	Riogen
K-X (Linde Type X)	K^+^, Na^+^	FAU (Faujasite)	1.3	Riogen
Na-Y (Linde Type Y)	Na^+^	FAU (Faujasite)	2.6	Riogen
K-Y (Linde Type Y)	K^+^, Na^+^	FAU (Faujasite)	2.6	Riogen
Na-A (Linde Type A)	Na^+^	LTA (Linde Type A)	1.0	Crosfield
K-A (Linde Type A)	K^+^, Na^+^	LTA (Linde Type A)	1.0	Sigma Aldrich
NH_4_-MOR (Mordenite)	NH_4_^+^	MOR (Mordenite)	10.0	Zeolyst
NH_4_-BEA-12 (Zeolite Beta)	NH_4_^+^	BEA (Zeolite Beta)	12.5	Zeolyst
NH_4_-BEA-19 (Zeolite Beta)	NH_4_^+^	BEA (Zeolite Beta)	19.0	Zeolyst
NH_4_-ZSM-5 (Zeolite Socony Mobil-5)	NH_4_^+^	MFI (Mobil Type Five)	40.0	Zeolyst
K-LTL (Linde Type L)	K^+^	LTL (Linde Type L)	3.1	Tosoh
CLPT (Clinoptilolite)	K^+^, Na^+^, Ca^2+^, Mg^2+^	HEU (Heulandite)	4.5	Natural zeolite (USA); Zeodex

* Structure type codes follow the convention established by the IUPAC Commission on Zeolite nomenclature [42,43].

**Table 2:** Potentiometric sensitivity of the sensors calculated between 10^−5^ and 10^−3^ M (±3 mV/dec).

Zeolite (Sensor)	Na^+^	K^+^	NH_4_^+^	Mg^2+^	Ca^2+^
Na-X (s1)	24	37	37	19	35
K-X (s2)	31	30	34	72	22
Na-Y (s3)	42	70	39	13	15
K-Y (s4)	38	56	42	19	15
Na-A (s5)	29	34	14	15	5
K-A (s6)	23	31	6	45	−6
NH4-MOR (s7)	47	57	34	28	12
NH4-BEA-12 (s8)	21	12	23	30	15
NH4-BEA-19 (s9)	30	35	27	29	8
NH4-ZSM-5 (s10)	18	42	20	11	6
K-LTL (s11)	33	58	35	53	20
CLPT (s12)	19	46	40	43	11

**Table 3:** Parameters of PLS regression models in 10-fold segmented cross-validation.

Parameter	Range, ppm	Slope	Offset	RMSECV, ppm	R^2^	MRE, %
**Total Hardness**	44–220	0.72	29	18	0.71	14
**Total Alkalinity**	14–75	0.79	12	9	0.76	18
**Na^+^**	2–61	0.81	3	5	0.80	30
**K^+^**	1.5–16.6	0.79	0.9	1.4	0.74	29
**Mg^2+^**	5–104	0.57	16	13	0.52	35
**Ca^2+^**	22–117	0.70	20	12.4	0.66	17

## Data Availability

Not applicable.

## Supplementary Materials

The following are available online at https://www.mdpi.com/article/10.3390/s21134471/s1, Table S1: An example of potentiometric responses of sensors (±3 mV/dec)., Table S2: An example of reference data.
