# Supplementary material for: A Pencil-Drawn Electronic Tongue for Environmental Applications
Source: Sensors (Basel). 2021 Jun 29;21(13):4471. doi: 10.3390/s21134471 (PMC8272086; doi:10.3390/s21134471)
Supplement: Supplementary file 1 [file sensors-21-04471-s001.zip › sensors-1206057-supplementary.pdf]

Supplementary Material

A Pencil-Drawn Electronic Tongue for Environmental Applications

Dmitry Kirsanov [1,2,*], Subhankar Mukherjee [3], Souvik Pal [3], Koustuv Ghosh [3], Nabarun Bhattacharyya [3], Rajib Bandyopadhyay [4], Martin Jendrlin [5], Aleksandar Radu [5], Vladimir Zholobenko [5], Monireh Dehabadi [1] and Andrey Legin [1,2]

[1] Institute of Chemistry, Mendeleev Center, St. Petersburg State University, Universitetskaya nab. 7/9, St Petersburg 199034, Russia; dehabadi16752@gmail.com (M.D.); a.legin@spbu.ru (A.L.)

[2] ITMO University, St Petersburg 197101, Kronversky pr. 49, Russia

[3] Agri and Environmental Electronics (AEE) Group, Centre for Development of Advanced Computing (C-DAC), Sector—V, Salt Lake, Kolkata 700091, India; subhankar.mukherjee@cdac.in (S.M.); souvipal@gmail.com (S.P.); koustuv02@gmail.com (K.G.); nabarun.bhattacharya@cdac.in (N.B.)

[4] Department of Instrumentation & Electronics Engg, Jadavpur University, Salt Lake Campus, Block LB, Sector III, Kolkata 700098, West Bengal, India; rajib.bandyopadhyay@jadavpuruniversity.in

[5] Lennard-Jones Laboratories, Birchall Centre, Keele University, Keele, Staffordshire ST5 5BG, UK; m.jendrlin@keele.ac.uk (M.J.); a.radu@keele.ac.uk (A.R.); v.l.zholobenko@keele.ac.uk (V.L.Z.)

Table S1. An example of potentiometric responses of sensors (±3 mV/dec).

	NaX	LTL	ZSM5	KX	BEA19	BEA12	K4	NaA	Na4	CLPT	MOR1	KA
sample1	297.5	304.0	318.1	294.7	305.1	281.2	302.1	294.5	314.3	291.9	347.9	29.9
sample2	300.1	305.8	318.6	292.1	305.5	279.8	302.9	292.4	312.7	290.2	350.6	50.0
sample3	302.5	307.3	311.1	300.4	303.3	288.1	309.1	300.9	318.8	297.5	336.5	60.1
sample4	306.1	310.2	313.4	302.8	307.1	289.9	309.9	302.6	317.9	290.8	337.6	56.7
sample5	298.8	309.1	313.2	306.0	301.4	281.6	308.4	299.5	317.3	297.6	317.4	91.6
sample6	302.3	310.0	312.6	304.9	305.9	287.6	311.6	301.8	320.8	298.9	327.7	49.5
sample7	302.6	308.8	315.6	301.6	305.2	289.9	308.7	301.8	318.9	290.2	344.4	77.7
sample8	306.9	306.4	312.0	298.7	305.9	286.9	305.5	304.8	316.2	288.8	347.4	33.7
sample9	298.5	307.7	316.5	305.5	301.7	287.6	309.7	307.2	322.9	304.8	330.9	38.8
sample10	301.4	314.0	315.5	304.6	303.6	288.7	312.6	303.8	323.4	304.3	333.4	94.1

Table S2. An example of reference data.

	Total Hardness	Total Alkalinity	SO4 = (ppm)	Cl (ppm)	Ca (ppm)	Mg (ppm)	Na (ppm)	K (ppm)	Cu (ppm)	Fe (ppm)
sample1	88,90	22,00	5,50	49,70	80,16	8,74	3,70	1,58	0,01	0,05
sample2	106,41	23,00	5,00	21,70	48,09	58,32	3,74	1,55	0,01	0,06
sample3	99,60	28,00	9,50	35,50	80,16	19,44	4,09	1,90	0,01	0,03
sample4	65,00	25,00	10,00	35,50	42,68	23,32	4,04	2,02	0,01	0,06
sample5	67,66	25,00	7,50	14,20	36,07	31,59	5,00	2,10	0,01	0,07
sample6	68,15	26,00	16,00	28,40	49,69	18,46	5,04	1,99	0,01	0,05
sample7	61,84	26,00	8,50	28,40	33,66	28,18	6,11	2,45	0,01	0,06
sample8	67,18	30,00	8,20	35,50	49,69	17,49	5,94	2,52	0,01	0,05
sample9	46,73	14,00	10,50	21,30	38,47	8,26	4,66	2,07	0,02	0,04
sample10	48,22	23,00	4,00	14,20	36,07	12,15	4,95	2,08	0,02	0,02
